# Obesity and eating behaviors in school children and adolescents –data from a cross sectional study from Bucharest, Romania

**DOI:** 10.1186/s12889-015-1569-9

**Published:** 2015-03-01

**Authors:** Carmen Gabriela Barbu, Monica Delia Teleman, Alice Ioana Albu, Anca Elena Sirbu, Sorina Carmen Martin, Adrian Bancescu, Simona Vasilica Fica

**Affiliations:** Carol Davila University of Medicine and Pharmacy Elias Hospital Endocrinology Discipline/Elias Hospital, 17 Marasti bvd, Bucharest, 7900 Romania; Department of Epidemiology, Carol Davila University of Medicine and Pharmacy Epidemiology Discipline/National Institute for Development and Research in Microbiology and Imunology “Cantacuzino” Bucharest, Spl. Independentei nr. 103, Sector 5, Code 050096 Bucharest, Romania

**Keywords:** Children obesity, Unhealthy eating behavior

## Abstract

**Background:**

Epidemiological data on obesity prevalence are scarce in Romanian population. Consequently, the aim of our study was to evaluate the prevalence of obesity and unhealthy behaviors among school children and adolescents from Bucharest, Romania.

**Methods:**

Cross-sectional study, 866 participants (53.2% girls, 46.8% boys, age range 6–18 years), selected by systematic sampling with probability-proportionate-to-size from all Bucharest’s schools.

Measurements: height, weight and a questionnaire to collect information about life style and eating behavior. Nutritional status was established based on World Health Organization recommendations (WHO), International Obesity Task Force (IOTF), Center for Diseases Control (USA-CDC) cut off values and local standards, respective.

**Results:**

The prevalence of overweight (including obese) and obesity alone based on different standards, was 31.6% and 11.4% (WHO), 24.6% and 6.2% respectively (IOTF), 25.2% and 10% (USA-CDC), 22.3% and 12.5% (local standards). When using local standards (weight only based), the obese subjects proportion among overweight children raised questions regarding the appropriateness of these standards. Overweight (including obese) prevalence was significantly higher among the boys versus girls: 36.2% vs. 27.6%, ( OR 1.5; 95% CI 1.12-2.03; p value = 0.006) and among the 6–10.9 years vs. 11–17.9 age group, (40.7% vs 26.6%). Almost all the participants (95%) reported at list one unhealthy eating behavior but no significant relationship was found with overweight or obesity only.

**Conclusions:**

This first epidemiological study of obesity prevalence in school children and adolescents showed that 11.4% of Bucharest’s children and adolescents were obese by WHO classification, 6.1% by IOTF cut off values and 10% by CDC classification. Younger children and the boys were more affected no matter which standard we used. In spite of unsignificant relationship to the adiposity status, our data showed a high prevalence of unhealthy eating behaviors reported by the participants. Particular aspects of the overweight versus obesity prevalence, after applying local standards, suggests that international recognized algorithms should be used for constant epidemiological evaluation instead of establishing local criteria.

## Background

Childhood overweight and obesity have reached epidemic proportions in most industrialized countries [1,2]; this aspect is mirrored by a similar phenomenon in the scientific papers on this topic, at least in some countries.

In 2004, according to IOTF criteria, it was estimated that ~10% of children worldwide aged 5–17 years were overweight and that 2–3% were obese, [3] with more than 40 million children under five being overweight in 2011, according to the WHO report [4].

Prevalence rates vary considerably between different regions and countries, from <5% in Africa and parts of Asia to >20% in Europe and >30% in the Americas and some countries in the Middle East [2,3,5].

Becoming obese earlier in life clearly amplifies certain health risks like hypertension, impaired glucose tolerance and type 2 diabetes, liver disease, and obstructive sleep apnea [6,7]. Obese children report a lower quality of life and demonstrate more negative self-perceptions, decreased self-worth, increased behavioral problems, and lower perceived cognitive ability [8]. Obesity in childhood is also a cause for insulin resistance and a significant predictor of adult obesity [7]. For children who remain obese into young adulthood, life expectancy can be shortened by as many as 20 years and all the associated co morbidities will increase the medical expenses [7,9]. Early prevention of childhood overweight is therefore very important but prevention strategy has to be supported by accurate epidemiologic data [1,2].

In spite of increasing concerns related to childhood obesity, when looking at an epidemiologic data map, we still found many regions or countries lacking high-quality data [10]. This is also the case in Romania, where regional reports were published only recently on this issue [11-14]. During the 1990s, tremendous socioeconomic changes took place in Romania and other Eastern European countries; consequently, we might expect important changes in the prevalence of childhood overweight and obesity, increasing the need for population studies or to update existing information. In Romania, we have data collected through the national school medical offices that are used to provide national reference cut-off values for weight categories in children. These values are subject to internal reports only (the last report was in 2007, unpublished data), and lacking proper methodology reference, are of limited use and do not allow comparisons to other countries. Therefore, we designed a study to evaluate the prevalence of overweight and obesity among school children and adolescents from Bucharest. From an economic point of view, Bucharest, the capital of Romania, is the most developed urban area, with the highest incomes and access to food, education, and medical care on average. On the other hand, the city is a melting pot of different lifestyles due to its citizens who have come from different regions or even abroad; it has also had increased economic polarization with huge discrepancies among the income of different population categories. Bucharest has 1.5 million inhabitants (8% of the total Romanian population in 2011), including 165,370 children and adolescents registered in the 2010–2011 school year, and no study was done for this population in the last three decades. All the statistic data provided are based on internal reports using particular local standards. This is the first study designed to evaluate the obesity prevalence among school children and adolescents in Bucharest, Romania meant to provide epidemiological data based on widely used references and to provide specific risk factors associated with increased adiposity in our population.

## Methods

### Ethics statement

Our research involved human participants and has been approved by the Elias Hospital ethics committee in September 2010. Written inform consent have been obtained from parents or legal guardian for all the children participating in the study. All clinical investigation has been conducted according to the principles expressed in the Declaration of Helsinki.

### Selection of subjects, recruitment, and approval

The target population consisted of all children registered in Bucharest’s schools in the 2010–2011 school year, from first grade in the primary school to twelfth grade in the high school. According to the city’s records, 165,370 pupils from 6 to 23 years of age were registered in Bucharest’s schools. In Romania, the school is mandatory for all children from first to twelfth grade; the minimum allowed age for school registration, by national rules is a minimum of 6 years for first grade. The maximum age of a registered student typically does not exceed 23 years but is frequently over 18 in eleventh and twelfth grades.

Based on a previous pilot study, the estimated prevalence of obesity was around 12% [15]. Taking this into consideration, a representative sample size of 1,108 subjects (including 10% to correct for non-responders) was selected by systematic sampling with probability-proportionate-to-size from a list of 270 schools from Bucharest. After participation approval was received from the selected schools (100% acceptance), two classes were randomly chosen from each of the 25 schools participating in the study according to the grade distribution (how many classes for each grade were registered in the whole city). These was meant to ensure the sample’s representativeness for age distribution. All the students from the selected classrooms were invited to participate in the study, and parents were asked to sign an inform consent form before the study was conducted.

### Data collection

Data were collected in May 2011 over a period of three weeks through clinical evaluation in the medical office of the school and questionnaire-based interview, intended to collect information about lifestyle, eating behavior, and family history.

Parents completed a questionnaire regarding their children’s height and weight at birth, the parents’ height and weight, brothers/sisters’ height and weight, if applicable, as well as some data about the medical history of the children and their relatives, which are not shown in present analysis.

Anthropometric data were collected by field teams, including school physicians, school nurses, and endocrinologists. Weight was measured in minimal clothing, without shoes, to the nearest 0.1 kg, with a calibrated mechanical step scale; height was measured to the nearest 0.5 cm in the same condition using a standard stadiometer from the school medical office.

Data related to food consumption and physical activity behaviors were collected through a questionnaire given out by the research team with respect to the number and distribution of meals per day, the number of meals in a fast food outlet, the time when the last meal of the day was eaten, the frequency of sweet snacks eaten at school, time spent daily on the television/computer, physical exercise, and types of daily food intake. For children up to 12 years old, the data were collected from parents, while children older than 12 years of age completed the questionnaire by themselves in the presence of a research team member.

### Definitions

Body mass index (BMI) was calculated from weight and height measurements, based on the following formula: BMI = weight (kg)/height (m)^2^. Overweight, obesity, and normal nutritional status were established based on the WHO 2007 recommendations [11,16], IOTF 2012 cutoff values [17,18], CDC [19], and after a comparison to local reference population normative data. For the local reference comparison, overweight was defined as a weight equal to or greater than the 90^th^ percentile for gender and age, and obesity as a weight equal to or greater than the 97^th^ percentile for gender and age. Normal weight in local standards was defined as a weight value greater than the 10^th^ and less than the 90^th^ percentile for gender and age. The reference population in local standards was Bucharest’s child and adolescent data reported from the school medical offices network in 2007 and analyzed by the Romanian National Institute of Health (unpublished data). Underweight was considered for all the subjects not classified in normal or overweight groups according to different definitions.

### Data analysis

WHO AnthroPlus software was used for assesing growth and adiposity status according to WHO recommendations [20], BMI for age grading for IOTF recommendation was done using the LMS growth [21] for Excel (Microsoft Office 2007). Data analysis was performed using the Statistical Program for Social Sciences for Windows (SPSS, Version 16). Descriptive summary measures of central tendency and frequency of the study variables were computed as appropriate. Bivariate categorical data were analyzed using Chi-Square and Fischer’s Exact Test, whichever appropriate. Continuous variables, which failed in the assumption of normality and homogeneity of variance, were compared across the groups by the Man-Whitney test. All statistical tests performed were 2- tailed and statistical significance was defined by a p value < 0.05.

## Results

A total of 1108 subjects were invited to participate to the study, reflecting the gender, age (defined by the class grade), geographical, and curricular type of the school (theoretical or vocational) distribution of the 165,370 pupils registered in the city’s schools. The reasons students did not participate in the study were as follows: (a) the subject was absent on the day of evaluation (106 subjects); (b) the parents did not agree with the study (8 for any kind of evaluation and 180 for the eating behavior and lifestyle evaluation only). We did not have records about the actual age distribution of the students registered in Bucharest, so of the 994 participants, we had to exclude 128 because they were over 18 years of age.

The analysis was performed on 866 children and adolescents (78% of 1,108) who were representative of the Bucharest school population (461 girls and 405 boys). The subjects older than 18 years were excluded from the study to be in conformity with the definition of a “child”.

In Table 1, the basic parameters of the study subjects are presented. The BMI ranged from 10 kg/sqm to 37 kg/sqm, with a standard deviation of +/− 3.9 and a normal distribution; percentile values by age and gender are shown in Table 2.

**Table 1:** **Main parameters in study subjects**

	**Boys**	**Girls**
**Main parameters**	**Mean (+/− SD)**	**Mean (+/− SD)**
**Age (years)**	12.6 (+/− 2.9)	11.8 (+/− 2.8)
**Height Z score (WHO 2007)**	0.13 (+/− 1.02)	0.15 (+/− 1.04)
**Weight Z score (WHO 2007)**	0.85 (+/− 1.26)	0.53 (+/− 1.33)
**BMI Z score (WHO 2007)**	0.12 (+/− 1.02)	−0.11 (+/−0.96)

**Table 2:** **BMI percentile by age and gender (School children: 6–17.9 years old)**

	**BMI percentiles**						**BMI percentiles**					
	**Boys**						**Girls**					
**Age (years)**	**3**	**10**	**50**	**90**	**95**	**97**	**3**	**10**	**50**	**90**	**95**	**97**
**6**	-	-	-	-	-	-	19.4	19.4	27.9	36.4	36.4	36.4
**7**	13.3	13.8	16.9	22.5	23.2	23.3	12.1	12.9	15.3	25.1	25.6	25.6
**8**	13.3	13.8	17.5	22.5	24.1	24.4	12.5	13.8	16.4	22.2	25.9	28.5
**9**	12.3	14.9	16.3	22.3	24.6	25.7	12.7	13.5	18.6	23.5	24.8	27.7
**10**	13.2	13.9	18.3	23.3	25.6	25.8	13.8	15.4	18.0	23.4	24.2	25.8
**11**	11.6	15.8	18.8	26.3	28.6	30.9	13.6	14.5	18.1	22.8	24.7	28.1
**12**	14.8	15.9	18.9	24.3	27.8	27.8	14.9	15.8	18.4	23.8	26.3	26.3
**13**	14.4	16.2	20.0	29.0	34.7	35.6	12.9	16.9	20.5	26.2	28.1	28.1
**14**	16.3	17.0	20.4	27.6	31.2	31.2	16.5	17.1	20.0	23.7	24.1	24.7
**15**	16.6	17.2	20.8	26.7	29.0	30.9	16.1	17.6	19.8	24.5	32.1	35.6
**16**	15.1	16.7	20.3	26.3	27.9	28.2	15.2	16.5	21.6	25.4	29.9	30.4
**17**	16.5	17.9	22.2	29.0	35.6	36.9	17.3	17.4	19.9	27.5	30.4	30.4

**Figure 1:**
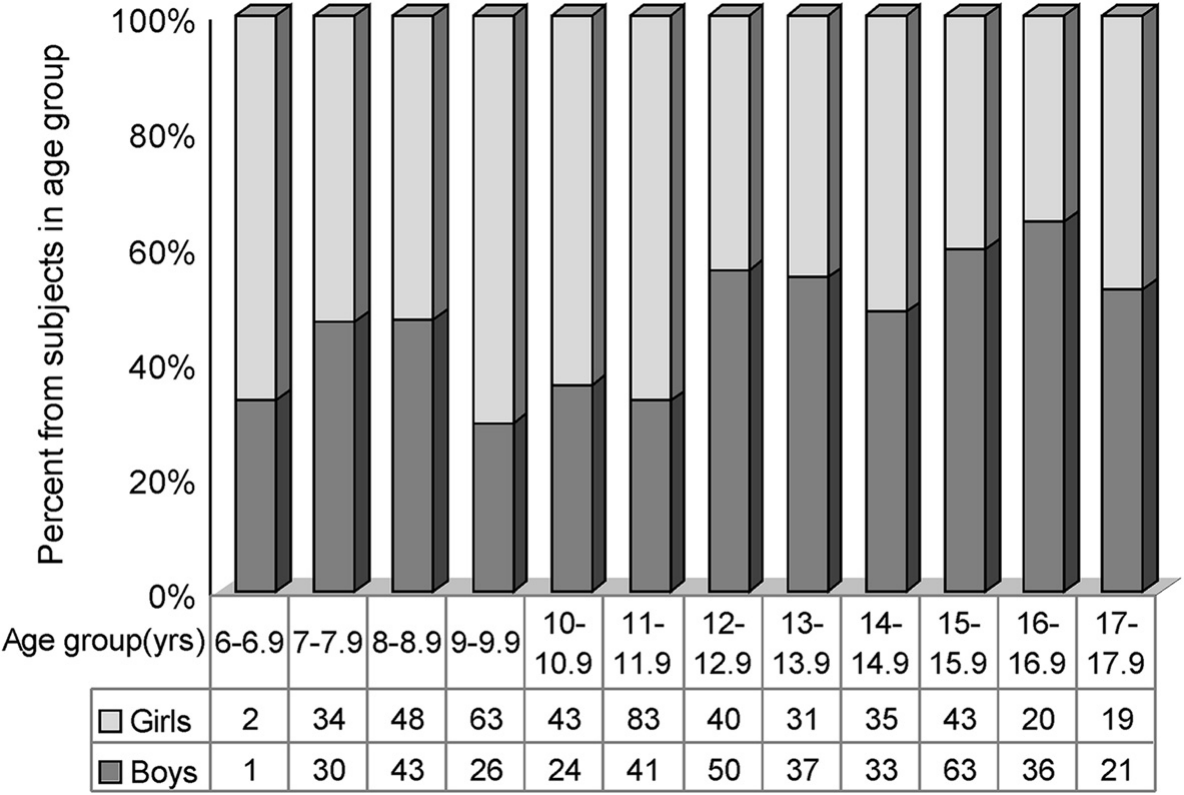
**Gender distribution of the study subjects represented as percentage by age.** The age group is 1 year range and is displayed below the columns. Below the age groups, absolute number of the subjects by gender are displayed.

The overall female/male ratio was 1.14/1 (53.2% girls, 46.8% boys), and the distribution of gender by age is shown in Figure 1. These gender ratios are similar to those revealed by the municipality school network data.

### Overweight and obesity prevalence

Regarding the evaluation of the adiposity status, we mainly used the WHO 2007 criteria, but we analyzed the data according to IOTF 2012, CDC 2000, and local standards used in the school network as well: we found that 31.6% of the children were obese or overweight, according to the WHO criteria, compared to 24.6% in the IOTF classification, 25.2% in CDC classification, and 22.3% after a comparison to local reference population (see Table 3; overweight excludes obese children). The obesity prevalence was found to be 11.4% according to the WHO definition, 6.2% by the IOTF criteria, 10% by the CDC criteria and 12.5% when compared to local normative data. Overweight (excluding obesity) prevalence revealed much closer values in WHO (20.2%), IOTF (18.4%), CDC (15.2%) classifications, with a surprising 9.8%, revealed by classification based on local normative data. Normal weight was found in almost two thirds of participants when using international classifications (67.9% by WHO, 64% by IOTF and 68.4% by CDC) and a higher percent (73.7%) when using local standards. Underweight was found in different percentages from 0.5 to 11.4% according to different criteria used (see Table 3).

**Table 3:** **Prevalence of BMI categories according to the WHO/IOTF/CDC/local standards definitions**

**Category of subjects**	**WHO standards based prevalence (%)**	**IOTF standards based prevalence (%)**	**CDC standards based prevalence (%)**	**Local standards based prevalence (%) (2007)**
**Obese: Total**	**11.4**	**6.2**	**10**	**12.5**
Obese: Boys	14.3	8	12.5	13.7
Obese: Girls	8.9	4.5	7.8	11.5
**Overweight** ^**a**^ **: Total**	**20.2**	**18.4**	**15.2**	**9.8**
Overweight^a^: Boys	21.9	21.1	17.5	8.6
Overweight^a^: Girls	18.7	16.2	13.2	10.8
**Normal weight: Total**	**67.9**	**64**	**68.4**	**73.7**
Normal weight: Boys	63.1	62	63.8	74.5
Normal weight: Girls	72.3	65.9	72.5	73.1
**Underweight: Total**	**0.5**	**11.4**	**6.4**	**4**
Underweight: Boys	0.7	8.9	6.2	3.2
Underweight: Girls	0.1	13.4	6.5	4.6

^a^: Excluding obese.

When analyzing the relationship between adiposity status and gender, we found a significantly higher percentage of boys in the WHO category of overweight (including obese) than girls: 36.2% vs. 27.6% (Chi^2^ OR 1.5; 95% CI 1.12–2.03; p value = 0.006). The IOTF and local standards classifications were similar (see Table 3).

For further analysis, we took into account only the WHO classification, which is recommended in clinical practice in Romania. Consequently, looking at the relationship between adiposity status and age, we grouped together the closest values of overweight and obese frequencies, resulting in four age groups. According to this analysis, the prevalence of subjects in the category of overweight (including obese) was significantly higher among those in the age group of 9–10.9 years, (43.8%), followed by children of 6–8.9 years, with 37.8% and 11–13.9 years (32%), and lower in older children of 14 –17.9 years, with 21.3%.

The data suggest that in our population, younger children (6–10.9 years old) are more likely to be overweight or obese (59.17% vs. 26.7%, p < 0.01) than their older colleagues (11–17.9 years).

### Relationships with eating behavior and physical exercise

The questionnaire applied was intended to collect data from children and parents about certain behavioral risk factors like the number and distribution of meals within 24 hours, the number and type of snacks and soft drinks served at school, the type and duration of physical exercise, and the time spent in front of the television/computer. Among the 866 children with available anthropometric data, only 686 properly completed the questionnaires and thus were able to include in the analysis. The reason children did not complete the questionnaire was the parent’s disapproval (180 subjects) and was mainly in the younger group (less than 12 years). The subgroup analyzed for the data collected through the questionnaire is not significantly different from the study group in terms of gender or BMI distribution.

*Number and distribution of meals over 24 hours*. Subjects were grouped according to the meals or snacks they consumed over 24 hours. Data analysis revealed a similar prevalence of overweight and obese children among the three groups segregated by the number of the meals: three, two or one meal per day (34% vs. 35.7% vs. 30.05%, respectively). Similar results where revealed when analyse was done according to the number of the snacks served per day: 36.4% overweight and obese children among those serving more than two snacks per day comparing to 33.3%, 36.5% and 39.6% among those serving two snacks, one snack or no snack at all, respectively.

*Time when the last meal of the day was served*: Surprisingly, 94.5% of the subjects reported the hour of the last meal being frequently later than 22:00 H, with a higher frequency, 98.8%, in younger children (6–13 years old.) versus 85.2% in older ones (14–17 years) (OR 14.4; 95% CI 6.1–35.9; p < 0.001). No gender-related differences were found and no relationship between this risk factor and the prevalence of overweight, including obesity, was found among study subjects.

*Meals served outside the home*: This was an average of 1.46 ± 2.01 during the week days and 1.28 ± 1.38 during the weekends; there was no correlation with the adiposity status of the subjects. No significant difference was found between boys and girls with respect to the number of meals served outside the home, but a positive trend with age was observed for students with more meals served outside.

*Fast food*: 9.9% reported one fast food meal weekly, while 74.3% occasionally and 15.9% were never served a fast food meal. No relationship was demonstrated between adiposity status and the frequency of eating in a fast food restaurant.

*Soft drinks:* 75% of the subjects reported daily consumption of soft drinks. There was no significant difference in soft drink consumption frequency and overweight prevalence

*Snacks served at school*: 53.5% reported being served a homemade sandwich, 7.2% fruits, 1.7% milk/yogurt, and 37.6% chocolate, pretzels, pastry, or candies.

*Snacks served at home*: 75.1% reported having home served snacks consisting of chocolate and candies in 90.7% of cases and homemade sweets in 9.3% of cases.

*Eating when watching television or in a standing position:* 90–99% acknowledged one of these behaviors as routine.

Neither the type of snacks or eating behavior, like eating when watching television, or in a standing position, was associated with increased BMI values.

*Daily physical exercises* were reported as curricular activities in school (1.4 hours ±0.98), extracurricular organized activities (1.55 hours. ± 2.89), and non-organized activities (1.83 hours ± 1.83) with no particular differences among boys and girls. At the opposite site, daily hours spent in front of TV/computer were 2.8 hours ± 2.48 with an increased trend with the age. None of these were correlated with increased weight or BMI values in our study subjects.

## Discussion

*Comparison to other Romanian studies.* Compared to the previously reported results of an 11.8% prevalence of overweight and obese Romanian pre-school children [22], our results suggests a higher overweight and obesity prevalence in school-age children than those under 4 years of age. In a similar age range, a previous pilot study conducted in Bucharest in 2004 demonstrated a similar prevalence among school-age children [15], sustaining a constant prevalence in the last years for this population. The overall 6.2% and 18.4% values found in our study for the IOTF 2012-based definition of obesity and overweight prevalence were similar to data reported in other regions of Romania in school children of a similar age. Thus, in the western part of Romania, a study conducted on school children (7–18 years old) from Timis county indicated an overall prevalence of 18.2% for overweight and 7.2% for obesity using the IOTF 2000 definition [13]. In Cluj county (the north-central part of Romania), 8.29% of the same-age school children were found to be obese and 12.84% overweight based on the 85^th^ and 95^th^ percentiles [12]. Results from the northeastern part of Romania (Iasi county) showed a prevalence of 16.6% overweight and 7.1% obesity among the studied school children (6–10 years old) based on the IOTF definition [14]. In comparison to the Romanian study using a similar edfinition of obesity [13], our data are derived exclusively from an urban population, which could explain the small difference, suggesting a higher prevalence of overweight and obesity among rural children; this needs to be clarified in a future study.

*Comparison to international data*. The prevalence rate of overweight, including obesity, in the representative sample of school children from Bucharest showed values comparable to the highest levels in European countries and the United States. The value of 31.6%, according to the WHO classification, and a respective 24.6%, based on IOTF standards, classifies the school population of Bucharest as among the most affected children populations from Europe [5]; the values are similar to those in southern Italy [23], to Greece (similar in girls, but worst in boys) and higher than the Czech Republic data [23] and almost all of the former Yugoslavian republics (except Yugoslavia itself, with a much closer value in terms of obesity in younger children) [23].

*Regarding the relationship to socioeconomic status*, different studies showed that certain population groups with greater access to high-energy diets (low-socioeconomic status in industrialized countries and high-socioeconomic status in developing countries) have an increased risk of being obese than their counterparts [24], which is reported in the Czech Republic as well [25], a country close to Romania but with a higher income. It can be very tempting to link the high prevalence of overweight and obesity in Bucharest’s school children and adolescents to higher levels of consumerism and a higher income than other cities of Romania, but similar values were reported in other regions of Romania, and a higher prevalence of obesity was also reported in rural areas associated with lower incomes [13]. As shown above in the article, Romania in general and Bucharest in particular experienced a rapid development in the last 22 years, but the total income is still low compared to developed countries, which will have to be analyzed in relationship to childhood obesity in future studies.

*The use of local cutoff points for adiposity status classification in the Romanian population* is questionable for several reasons: the lack of population-specific extensive epidemiological data, and the chance of losing the opportunity for international comparison; in clinical practice, it is recommended to use the WHO reference. On the other hand, we have a national network of school-based medical offices collecting data on some anthropometric parameters in school children at certain ages. The analysis of school-reported data for children in Bucharest, performed in 2007 by the National Institute of Health, provided the reference and local standards used in this study’s analysis. Looking at the results, the IOTF standards showed a lower prevalence of overweight including obesity than the WHO criteria (24.6% vs. 31.6%), which was already revealed by previous analyses [26]. The local reference population weight-based classification revealed a prevalence of obesity similar to the one found by the WHO criteria, but surprisingly, comparing the obesity and overweight prevalence separately, the data analysis revealed an unexpected below 1 ratio between overweight and obese subjects (9.8% to 12.5%), following local reference cutoff points, which is explained by the use of “only weight” cutoff values; this also raised questions regarding the accuracy of the data collected through the school medicine network.

Our data showed a *higher prevalence of overweight (including obese) among boys (*36.2% vs. 27.6%); this aspect is frequently reported both in Romanian and international reports even they have used different definitions or studied different age ranges [2,3,5,12-14]. This might be linked to the reduced number of meals in males but since the association was not significant could be just a working hypothesis.

As reported in other populations as well [3,5,8,27], the *most affected age groups were the younger ones* (40.77% in prepubertal age vs. 26.6% in pubertal and post pubertal ages) which might be explained either by the attenuation of the positive energy, balanced during puberty (not sustained by the lower prevalence in the post adolescence group), or a possible positive trend of increased weight across generations due to exposure to environmental factors earlier in life.

*Unhealthy eating behaviors* (risk factors for obesity) like the last meal of the day being served later than 22:00Hr, the number of meals served outside the home, fast food meals, the type of snacks served at school or at home, eating when watching television or in a standing position, were not related to an increased prevalence of overweight (including obesity) in study subjects. We found an almost 100% frequency of unhealthy eating behavior, like eating late at night, even in very young children, with no differences across BMI categories. This might suggest that a lack of obesity in these conditions is only the consequence of inherited protective factors—a hypothesis that will need further analysis. On the other hand, these findings could be explained either by the increased calories found in homemade food or by responders’ bias (due to the negative image associated with unhealthy eating behavior, resulting in a trend toward denying it).

Physical activity, which was not queried in detail in the questions, was reduced in almost all subjects to a minimum (less than 2 hours per week) especially in adolescents, in concordance with other international findings [28]. Our study addresses only two aspects predicting sedentary behavior, the daily television/computer hours and weekly physical activity; nevertheless, the correspondence between different activities and sedentary behavior was found to be somewhat unreliable in a study addressing twelve different aspects predicting sedentary behavior [29], which might explain the lack of correlation to obesity.

To our knowledge, this is the first study to provide a descriptive epidemiology of adiposity status based on the most frequent international recommendations as well as regarding a variety of eating behaviors in 6–18-year-old children and adolescents from Bucharest.

The sample size and heterogenity in location and curricular type of schools are key strengths of our study. On the other hand, one may say that the exclusion of the participants who were over 18 years of age from the final analysis could have affected the representativeness of the sample. The lack of the records about the actual age distribution of the students registered in Bucharest for a certain class grade imposed a limitation on the sample selection; therefore, from the 994 participants, we had to exclude 128 because they were over 18 years of age. Consequently, the non-response rate was actually 10.2%, which is similar to the estimated one of 10%.

Several limitations are acknowledged. The LMS values were not optimal due to a increased oscillation in the percentiles values across the age groups. We appreciate it

was the effect of absenteism in the classes selected to enter the study, children or parents refusal to participate in the study and exclusion of subjects older than 17 years on the representativity of each age group. It has to be mentioned that representativity for age groups was planned to be asured by the school grades representativity. The cross-sectional design did not allow us to investigate causal relationships between the behaviors and overweight and might explain the lack of a significant association between excess adiposity and eating behaviors that are considered unhealthy. However, we obtained information on the types of behaviors children engaged in, and one particular aspect is the increased extension of the unhealthy eating behaviors among all children and adolescents. The nonresponse rate for the eating behaviors and physical exercise questionnaire was higher than nutrtional status evaluation; only 686 subjects completed it of the 866 who were younger than 18 years of age. According to our data, there were no significant differences between the BMI of the non-responders and the responders. Another limitation might be the different sources of information: subjects older than 12 years of age completed the questionnaire by themselves and the younger ones had their parents answer the questions.

## Conclusions

The present findings indicate a 31.6% prevalence of overweight (including obesity), among Bucharest’s school children, according to the WHO criteria, with an 11.4% prevalence of obesity. IOTF reference standards showed, as expected, a lower prevalence of 24.6% in the category of overweight (including obesity) and a 6.2% prevalence rate of obesity. The WHO values were similar to the CDC classification (25.2% overweight including obesity and 10% obesity alone). Different classifications allowed a comparison to other population data, putting our study children and adolescents among the most affected in the European population as a whole. Boys were more affected than girls (36.2% vs. 27.6%), no matter which classifications were used; a higher prevalence of overweight including obesity (40.77% vs. 26.6%), was found in younger children (6–12 vs.12-18 years age groups), which might be the result of some generalized unhealthy eating habits, like a late hour of intake for the last meal of the day. The unusual distribution of overweight versus obesity prevalence, after applying local reference standards, suggests that international recognized algorithms should be used for constant epidemiological evaluation instead of establishing local criteria or using self-reported data from school networks. All these aspects also underlie the importance of good epidemiologic data in customizing educational programs focused on children and parents’ education on a healthy lifestyle.

## Abbreviations

WHO: World Health Organization recommendation
IOTF: International Obesity Task Force
USA-CDC: Unitated States of America- Center for Diseases Control
OR: Odds ratio
CI: Confidence interval
BMI: Body mass index
Hr: Hour
